# The Role of Habitat, Climate, and Space in the Species and Traits Variation of Phytoplankton in Large Cascade Reservoirs

**DOI:** 10.1002/ece3.72645

**Published:** 2026-01-14

**Authors:** Idelina Gomes da Silva, Bárbara Dunck

**Affiliations:** ^1^ Laboratório de Ecologia de Produtores Primários Universidade Federal do Pará Belém Pará Brazil; ^2^ Laboratório de Ecologia Aquática e Aquicultura Tropical, Instituto Socioambiental e dos Recursos Hídricos Universidade Federal Rural da Amazônia Belém Pará Brazil

**Keywords:** environmental filters, functional traits, hydroclimatic, nestedness, turnover

## Abstract

The construction of cascade dams on large rivers reduces river connectivity, increases environmental homogenization by altering the longitudinal processes responsible for high biodiversity, and may restrict the establishment and survival of species without functional traits in these locations. Our study evaluated phytoplankton beta diversity regarding taxonomic and functional facets in the seven cascade reservoirs of the River Tocantins (Brazil), along 1500 km in length, between 2006 and 2014. We tested whether (1) younger reservoir age leads to higher values of taxonomic and functional phytoplankton beta diversity than the others; (2) whether there is a positive correlation between functional and taxonomic phytoplankton beta diversity in cascade reservoirs; (3) and the taxonomic and functional beta diversity of phytoplankton is more strongly determined by spatial mechanisms than by local hydroclimatic and environmental conditions, with a stronger explanation expected for functional beta diversity. We demonstrated that younger and more distant reservoirs present higher values of taxonomic and functional phytoplankton beta diversity, and that reservoirs in the intermediate regions of the cascade, close to each other and with greater environmental similarity, presented taxonomic and functional homogenization of phytoplankton. Finally, we found that spatial variables were more explanatory of the variation in species and functional characteristics of phytoplankton communities compared to local environmental and hydroclimatic variables.

## Graphical abstract

Phytoplankton beta diversity along cascade reservoirs of the Tocantins River is shaped by reservoir age, spatial arrangement, and environmental conditions. Younger, distant reservoirs show higher diversity, while intermediate ones tend toward homogenization. Environmental and spatial drivers differentially influence taxonomic and functional composition.

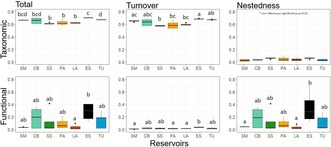

## 1 Introduction

Economic development and population growth are increasing anthropogenic pressure on global water resources (Cooley et al. 2021). Pollution, channeling, transposition, and construction of dams are the main agents promoting changes in freshwater ecosystems (Rosenberg et al. 2000). Human activities like these accelerate the decline of biodiversity on local, regional, and global scales (Chapin et al. 2000; Dudgeon et al. 2005; IUCN 2009). The implementation of dams in tropical environments, for example, promotes hydrological changes with serious consequences for biodiversity (Grill et al. 2019).

This anthropogenic pressure on water resources not only accelerates the decline of biodiversity, but it also poses significant challenges to the preservation of ecological communities. These impacts alter environmental functionality regimes, directly affecting the species present (Ganuza et al. 2022; Ferreira et al. 2024). Studying differences in species composition between different habitats over time and space reveals the mechanisms that structure communities (Meynard et al. 2011; Gutiérrez‐Cánovas et al. 2013). In this scenario, beta diversity, including its taxonomic (species variability) and functional (functional trait variability) facets, offers complementary perspectives by which to understand the structure and functioning of ecological communities. In space, it allows the identification of patterns associated with environmental gradients, heterogeneity, and connectivity; in time, it highlights changes linked to disturbances, succession, and colonization dynamics. In this context, although two communities may have similar taxonomic diversity, spatially or temporally, their functional traits may differ significantly (Mouillot et al. 2013) and thus influence the response of ecosystems to disturbances such as the installation of cascade dams (Mouillot et al. 2005; Petchey and Gaston 2006; Mouchet et al. 2010).

Multiple dams along a river promote significant loss of species and functional traits due to the cumulative effects of habitat fragmentation and changes in environmental conditions (Ward and Stanford 1983; Barbosa et al. 1999; Lu et al. 2020). Changes include the formation of physicochemical environmental gradients, such as the gradual reduction of nutrients downstream (Maavara et al. 2020; Zhao et al. 2024), and reduction of turbidity with consequent increase in the photic zone (Shen et al. 2022). These variables directly interfere with the survival of phytoplankton species, requiring suitability for such environments. However, spatial processes such as dispersal limitation, hydrological connectivity, and the position within the reservoir cascade often structure communities more strongly than local environmental filters in regulated river systems (Leibold et al. 2004; Heino et al. 2015).

Along the longitudinal axis of rivers, especially over large stretches, climatic gradients also result from variations in precipitation, solar radiation and air temperatures (Mesquita et al. 2020; Domingues and da Rocha 2022). This climate variability influences the hydrology of rivers, inducing spatial and temporal differences in flows and local environmental variables (Liang et al. 2020). For example, reductions in precipitation and rising air temperatures could reduce reservoir water volumes and increase nutrient concentrations. This would change species distributions, and species with functional traits suited to this situation would dominate (Heino and Alahuhta 2015; Wiegand et al. 2021; Zhou et al. 2022).

Species distribution in reservoirs is influenced not only by environmental and climatic gradients, but also by age. Younger reservoirs undergo early stages of ecological succession, characterized by high rates of colonization and extinction (da Silva et al. 2020), common features in undammed rivers where there is continuous replacement of species (Vannote et al. 1980). Initially, in the formation of reservoirs, the water column expands and incorporates different environments (aquatic and terrestrial). In this phase, opportunistic species with broad ecological tolerance are favored, while at the same time reducing the area available for those with more restricted niches (Agostinho et al. 2016; Souza et al. 2016). Furthermore, due to their high abundance and ease of dispersion, microorganisms spread rapidly in these environments (Astorga et al. 2012; Wu et al. 2019). In this context, niche‐based processes take on the role of environmental filters in reservoirs, promoting the gradual replacement of species and increasing extinction (Clavero and Hermoso 2011; Bozelli et al. 2015). Factors that drive both colonization and extinction can intensify species turnover, highlighting the ecological dynamics that occur (Soininen 2010; Nuvoloni et al. 2016). In older reservoirs, although this process is not yet fully understood (Juracek 2015), this dynamic is different, presenting totally lentic characteristics and greater environmental homogeneity, with implications for species composition and biotic homogeneity (Clavero and Hermoso 2011; Petsch 2016; Castro et al. 2021).

Environmental similarity between reservoirs and their geographic proximity also contributes to greater environmental and biotic homogeneity (Soininen et al. 2007; Wetzel et al. 2012; Graco‐Roza et al. 2022). The distances between them may favor species with functional traits that allow them to disperse and colonize these environments (Leibold et al. 2004; Heino et al. 2015; Lansac‐Tôha et al. 2021), as spatial factors are important in the succession and dispersal of species. Thus, changes caused by cascade dams can impact both taxonomic and functional diversity, since only species with suitable traits survive the new conditions generated by the impacts, resulting in a positive correlation between taxonomic and functional beta diversity (Heino 2008; Gallardo et al. 2011). However, the strength of this relationship is not trivial, as it depends on the degree of functional redundancy within communities (Várbíró et al. 2017). If these habitat filters are very strong or in the absence of niche filters, low functional dissimilarity may occur, in which case there will be a negative relationship between taxonomic and functional diversity.(Bêche and Statzner 2009; Villéger et al. 2012).

Studies on the dynamics of phytoplankton beta diversity in cascade reservoir systems are incipient. Some of them have evaluated how spatial and temporal gradients alter the distribution patterns of the phytoplankton community (Barbosa et al. 1999; Kumar et al. 2025). They point to the euphotic zone, suspended solids and nutrients as the main factors driving variations in phytoplankton functional groups between reservoirs (Engel et al. 2019; Shen et al. 2022). Some studies indicate that the increase in the number of downstream reservoirs and greater hydraulic stability explain the predominance of some groups as well as the reductions in richness and functional diversity (Da Silva et al. 2005; Bortolini et al. 2020; Resende et al. 2022), in addition to demonstrating that increasing retention time increases the abundance of phytoplankton (Luo et al. 2025). However, none of these studies evaluated how hydroclimatic gradients interfere with the composition of species and functional traits in phytoplankton communities. Highlighting these traits is particularly relevant, since the functional attributes of phytoplankton, especially those related to cyanobacteria and microalgae, play a key role in maintaining ecosystem processes in reservoirs, such as primary productivity and nutrient cycling (Tilman et al. 2014; Naselli‐Flores and Padisák 2022; Liu et al. 2024).

The taxonomic and functional diversity of phytoplankton is ecologically significant because it supports fundamental processes such as primary production, energy flow, and biogeochemical cycles (Delille et al. 2005; Taucher et al. 2022). Different functional groups of algae respond differently to environmental and hydrological gradients, directly impacting ecosystem stability and resilience (Litchman et al. 2010; Silva, Rodrigues, et al. 2025). Therefore, it is crucial to understand how the complexity of a system with multiple dams of different ages, in extensive areas with local hydrological, climatic and environmental gradients, interferes with phytoplankton beta diversity. Such knowledge not only improves water resource management in the basin but also supports actions to maintain the functioning of these ecosystems. In this context, this study evaluated how hydroclimatic, physical–chemical and spatial gradients interfere with phytoplankton beta diversity in cascade reservoirs along 1500 km of a large river in Brazil. Unlike previous studies, we integrate taxonomic and functional dimensions with multiple predictor variables, addressing a critical knowledge gap on how environmental, spatial and hydroclimatic drivers jointly shape community structure. This combined approach highlights the novelty and relevance of our study in advancing the understanding of biodiversity patterns in regulated river systems.

According to the premises presented, and considering that the reservoir at the end of the cascade is the youngest, while the reservoirs in the middle are spatially closer and environmentally more similar (Silva et al. 2025) we predict that: (H1) The youngest reservoir located at the end of the cascade will present higher values of taxonomic and functional beta diversity of phytoplankton, while the reservoirs in the middle, environmentally similar and spatially close, present a more similar composition, with less taxonomic and functional variation (H2). There will be a positive correlation in the functional and taxonomic beta diversity of phytoplankton in the cascade reservoirs (H3). The taxonomic and functional beta diversity of phytoplankton is more strongly determined by spatial mechanisms than by local hydroclimatic and environmental conditions, with a stronger explanation expected for functional beta diversity.

## 2 Methods

### 2.1 Study Area

**FIGURE 1:**
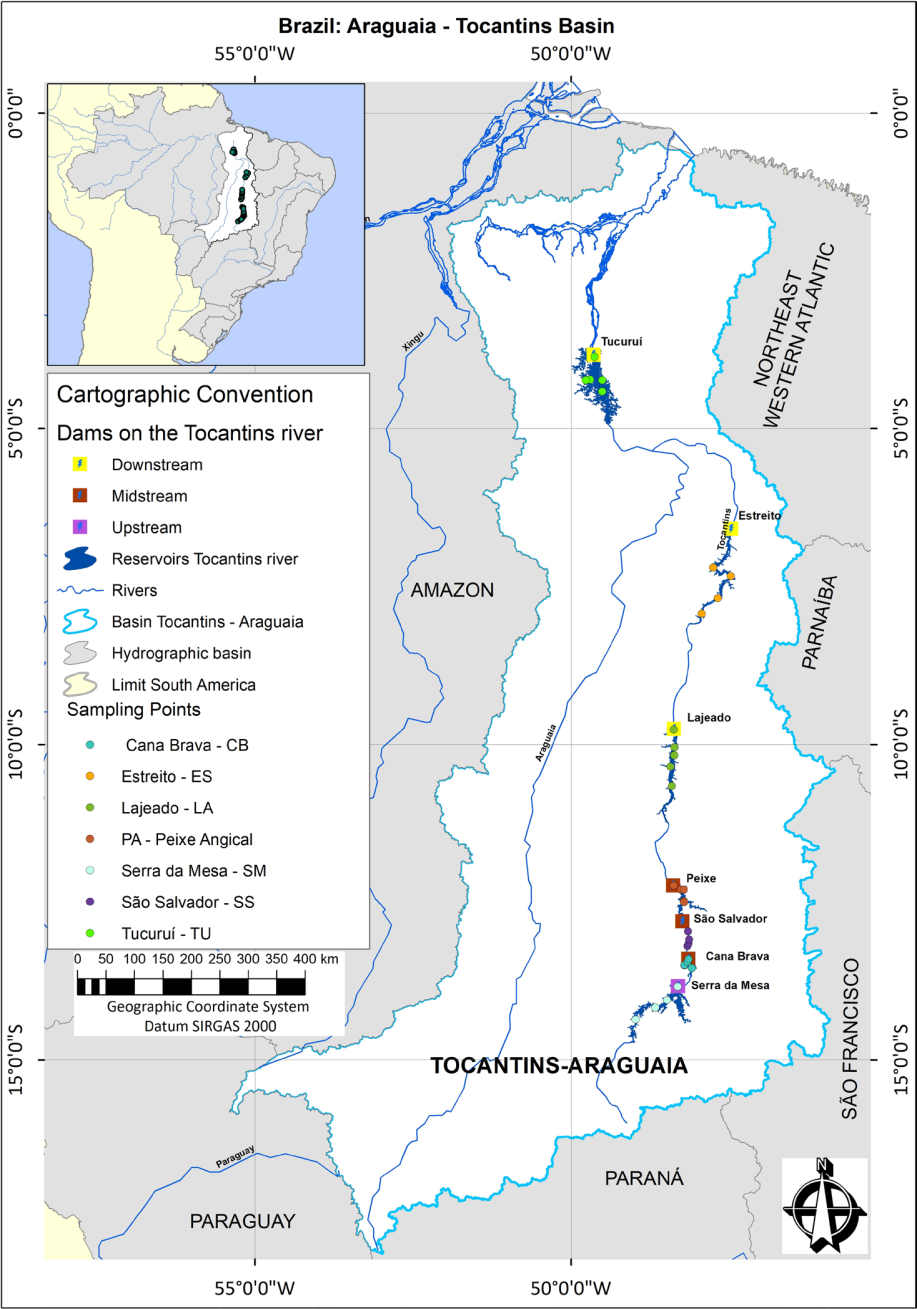
Map the location of sampling points in the cascade reservoirs on the Tocantins River.

The Tocantins River presents hydroclimatic and local environmental variability along its length. The installation of seven large cascading reservoirs along this route has reduced river connectivity and accentuated the hydrological and physical–chemical gradients of the water. Hydrological variability is promoted by differences in flow rates, which naturally increase from upstream to downstream in the longitudinal direction (Silva et al. 2025), but which are controlled according to the type of operation of the hydroelectric plants. In addition, there is climate variability that changes as longitudes decrease toward the equator. These spatial differences have been responsible for differences in climate factors such as precipitation, solar radiation, evaporation and air temperatures. Along the hydrographic basin, two distinct biomes are formed: In the South, the Cerrado/Savanna biome predominates, and in the North, the Amazon biome/dense rainforest prevails, both with considerable impacts from agriculture and mining (Costa et al. 2003; Pelicice et al. 2021; Swanson and Bohlman 2021). In this context, in the Tocantins River there are hydroclimatic gradients, different forest formations and land uses, dams and physical–chemical water gradients. The study area comprises the seven large reservoirs installed on the Tocantins River (Figure 1; Table 1).

**TABLE 1:** Hydrological details of the seven Tocantins River cascade reservoirs. Type of operation (0 = run‐of‐river and 1 = accumulation).

Hydrological details	Serra Mesa	Cana Brava	São Salvador	Peixe Angical	Lajeado	Estreito	Tucuruí
Retention time (days)	760	28	12	18	24	16	50
Average depth (m)	50.5	31.2	25	25	36	19.7	68
Type of plant operation	1	0	0	1	0	0	1
Position in the cascade	1	2	3	4	5	6	7
Area (km^2^)	1784	139	104	294	630	434	2850
Distance from the reservoir upstream (km)	0	51	67	65	274	365	389
Volume (bi m^3^)	54.4	2.36	0.043	2.7	5.19	5.4	43
Start of filling (year)	1998	2002	2009	2006	2001	2011	1984

### 2.2 Sampling

Sampling was carried out in seven reservoirs installed in cascades on the Tocantins River. Five sampling points were selected in each of the reservoirs, in fully lentic environments, close to the dams, between 2006 and 2014. Four campaigns were conducted per sampling point during the rainy season to avoid seasonality effects (*n* = 140). Serra da Mesa (Jan/Mar 2006–2007), Cana Brava (Jan/Mar 2012–2013), São Salvador (Jan/Mar 2011–2012), Peixe Angical (Feb/Apr 2012; Jan/Mar 2013), Lajeado (Oct/Dec 2013; Mar/May 2014), Estreito (Jan/Apr 2011; Jan/Mar 2012), and Tucuruí (Jan/Mar 2012; Oct/Nov 2012).

### 2.3 Hydroclimatic and Environmental Variables

Hydrological variables, hydraulic residence time (days), depth (m), and volume (m^3^/s) were obtained from technical reports (IBAMA). In the field, pH, conductivity (μS/cm), dissolved oxygen (mg/L), turbidity (NTU), and temperature (°C) were measured with a multiparameter probe YSI 6920. Water samples for total and dissolved nutrients were preserved on ice and analyzed in the laboratory: total nitrogen (Koroleff 1978; Mackereth et al. 1978), total phosphorus (colorimetry; Chapman and Pratt 1961), nitrate (cadmium reduction), and ammonium (phenol method; APHA 2005).

Precipitation data were obtained from seven rainfall stations (https://www.snirh.gov.br/hidroweb). The historical series of air temperature and global solar radiation were obtained from seven meteorological stations (https://portal.inmet.gov.br/dadoshistoricos). Flow that reaches a hydroelectric project or a hydraulic structure and average monthly net evaporation rate (m^3^/s) calculated for each reservoir (https://www.ana.gov.br/sar/sin/b_tocantins and https://metadados.snirh.gov.br/geonetwork). The geographic coordinates for the spatial data were obtained at each of the sampling points with the help of a GPS (Global Positioning System). All variables used in this study are listed in Table 2.

**TABLE 2:** Summary of annual averages of hydroclimatic (Hydro) and local environmental (physical and chemical) variables, their codes and descriptions used in this study.

Variables					
Codes	Unid	Description	Average	Min	Max
**Hydroclimatic**		**Hydroclimatic variables**			
HRT	days	Hydraulic Retention Time	27	12	760
Depth^a^	m	Depth	30	3	72
Flow	m^3^/s	Flow Rate	2.735	261	5.786
Prec	mm	Precipitation	103	0	499
Rad	MJ/m^2^	Solar Radiation	17	16	20
TempMax	ºC	Maximum air temperature	31	25	35
TempMin	ºC	Minimum air temperature	24	16	30
EVP	m^3^/s	Evaporation	6	−6	46
**Local environmental**		**Physical‐chemical**			
TN^a^	mg/L	Total Nitrogen	0.289	0.025	0.972
NH_3_^−^	mg/L	Nitrate	0.095	< 0.000	0.500
NH_4_^+^	mg/L	Ammonium	0.082	< 0.000	0.360
TP	mg/L	Total Phosphorus	0.014	< 0.000	0.048
Cond	μS/cm	Conductivity	61.507	32.000	99.000
pH	—	pH	7.466	6.700	8.540
DO	mg/L	Dissolved Oxygen	6	5	9
Turb	NTU	Turbidity	5	0.6	35
Temp	ºC	Temperature	29	26	32

^a^ Variables that presented multicollinearity and were removed from subsequent analyses.

### 2.4 Sampling and Analysis of Phytoplankton and Its Functional Traits

Phytoplankton samples were collected at 20 cm depth in the limnetic zone, fixed with 1% acetic Lugol's solution, and counted using the Utermöhl method (Utermöhl 1958), under an inverted microscope (Leica DM IL); density was determined following APHA (2017) guidelines.

In this study we evaluated two functional traits of phytoplankton species. They are the functional traits of the type response to environmental gradients, namely: Life forms (Unicellular, Filament, Colonial and Coenobic; Crossetti and de M. Bicudo 2008); and adaptation to flotation (Mucilage, Aerotope, Flagellum, Processes and Silica; Reynolds 2006). They were obtained from databases and published articles or from online databases (Bicudo and Menezes 2006; Spaulding et al. 2021; Guiry and Guiry 2023). Among the traits related to buoyancy, mucilage increases the surface‐to‐volume ratio and reduces cell density, which decreases sedimentation rates and favors permanence in the euphotic zone and structures called processes increase cell surface and volume, reducing density and, consequently, sedimentation (Padisák et al. 2003). Aerotope provides greater buoyancy and permanence in the water column, reducing cell density; the flagellum facilitates resource acquisition and movement, preventing sedimentation, In contrast, the presence of silica increases density proportionally to the sedimentation rate (Huszar and Reynolds 1997; Kruk 2002; Kruk et al. 2010). Regarding life form traits, unicellular cells facilitate resource acquisition and exhibit resistance to sedimentation; filamentous and colonial forms are also associated with resource acquisition (Litchman and Klausmeier 2008), while coenobium is related to depth self‐regulation (Reynolds 2006).

### 2.5 Data Analysis

The data matrices in this study were composed of the average of the annual values for all environmental parameters and the species density matrix. The averages of these values were performed to avoid temporal effects in the analyzes and totaled a sample size of *n* = 34. The species density matrix was transformed into a species presence and absence matrix for subsequent analyzes.

To calculate taxonomic beta diversity, we used the species presence and absence matrix and the Jaccard pairwise dissimilarity index. To calculate functional beta diversity, we used the species presence and absence matrix and the species functional trait matrix (Legendre and Legendre 2012). We performed a Principal Coordinates Analysis (PCoA) using a Gower functional distance matrix applied to the phytoplankton functional traits matrix (da Costa Lobato et al. 2015). The first two axes of the PCoA and species occurrence data were used to calculate total functional beta diversity, as well as the turnover and nestedness components (Baselga 2010). Total beta diversity was calculated and partitioned into turnover and nestedness (Baselga 2012).

To verify the differences in the values of total taxonomic and functional beta diversity, turnover and nestedness between reservoirs and between the different regions (beginning, middle and end), regarding the first hypothesis of our study, we performed analysis of variance (ANOVA) with the a posteriori Tukey test. The Shapiro–Wilk and Bartlett tests were used to verify the normality of the residuals and homogeneity of the variance. The components of beta diversity were the response variables, and the reservoirs and regions were treated as the categorical predictor variables. We performed a Permutational Multivariate Analysis of Variance (PERMANOVA—Anderson 2017) to compare species abundance across reservoirs. To visualize these differences, we then performed a Principal Coordinates Analysis (PCoA) based on Bray Curtis dissimilarity distances using the species abundance matrix standardized by the Hellinger method in each of the reservoirs. To test our second hypothesis and evaluate the correlation between functional and taxonomic beta diversity and its components, we performed the Mantel test with 9999 permutations (Legendre and Fortin 2010; Legendre et al. 2015).

To quantify the relative roles of local, hydroclimatic, and spatial environmental mechanisms for each facet of beta diversity and its components, related to our third hypothesis, we performed six variance partitioning analyzes (RDAp—Borcard et al. 1992). The dataset formed for this analysis included response variables composed of taxonomic beta diversity (total, turnover, and nestedness) and functional beta diversity (total, turnover, and nestedness), and the predictor variables were composed of local environmental variables with water physicochemical characteristics, hydroclimatic and spatial (31 eigenvectors) characteristics. We tested the significance of the pure fractions using ANOVA at a significance level of *α* = 0.05. Before proceeding with the RDAp analysis, we first checked for multicollinearity among the predictor variables and removed those with a variance inflation factor > 5 (O'Brien 2007). We sequentially performed variable selection, by forward selection, to find more parsimonious models for the analyzes of interaction between biotic and abiotic data. This selection was performed with two stopping criteria: significance level and the adjusted coefficient of determination (adjusted *R* ^2^; Blanchet et al. 2008) and performed separately for each abiotic data set and by response variable.

To represent directional spatial patterns associated with the unidirectional flow of water in the cascade reservoirs, we applied the Asymmetric Eigenvector Maps (AEM) analysis (Blanchet et al. 2008). Hydrological connectivity and geographic location, follow the downstream flow direction. Then, we used the geographical coordinates (latitude and longitude) of each reservoir to represent the spatial arrangement of the system. Using this information, we generated a directional neighborhood list through graph‐based methods that reflect the cascade's flow structure. Only eigenvectors associated with positive eigenvalues were retained, ensuring that the modeled spatial structure corresponds to the positive variance of biological data.

All analyzes were performed in RStudio (R Core Team 2020). To calculate taxonomic beta diversity, we used the beta.pair function, while for functional beta diversity we used the functional.beta.pair function, both from the betapart package (Baselga and Orme 2012). For the multicollinearity test we used the vifstep function from the USDM package (Naimi et al. 2014). We used the following functions from the vegan package (Oksanen 2019) for: variable selection (ordistep), spatial vectors (pcnm), Mantel (mantelI), RDAp analysis (varpart). The connectivity matrix was then used to generate asymmetric spatial eigenvectors using the adespatial R package (Dray et al. 2025).

## 3 Results

### 3.1 Beta Diversity Reveals Contrasting Patterns Between Upstream, Midstream, and Downstream Reservoirs

**FIGURE 2:**
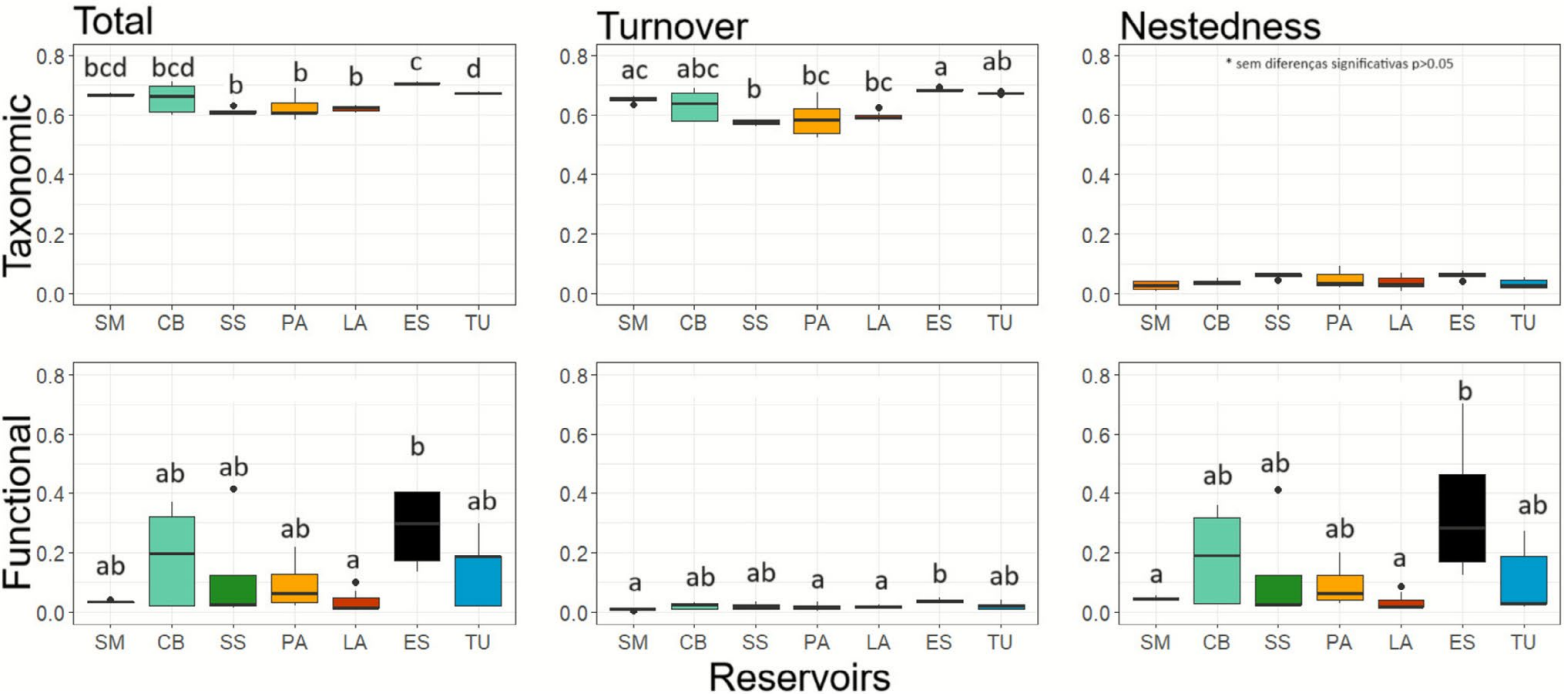
Phytoplankton taxonomic and functional beta diversity (total, turnover, and nestedness) among different reservoirs along the cascade. Different letters indicate significant differences (*p* < 0.05). The horizontal line in the box represents the median.

**FIGURE 3:**
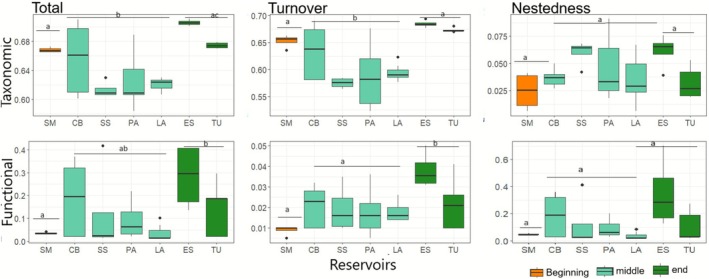
Representation of the spatial distribution of values for each component of beta diversity (total, turnover, and nestedness) in the taxonomic and functional facets, with significance test by longitudinal regions of the cascade (beginning, middle, and end). Different letters indicate significant differences (*p* < 0.05). The horizontal line in the box represents the median.

In the total taxonomic beta diversity, we observed a variation from 0.58 to 0.70. The ES reservoir, at the end of the cascade (newly formed), presented significantly higher values (*F*_(6,27)_ = 7.972; *p* = 0.010), while LA, PA and SS presented the lowest values (*F*_(6,27)_ = 7.97; *p* = 0.023). The taxonomic turnover presented variations from 0.57 to 0.69, with lower values in the median region of the cascade, represented mainly by the lower values of SS; thus, the middle of the cascade was different from the beginning (*F*_(6,27)_ = 9.43; *p* = 0.001) and the end (*F*_(6,27)_ = 8.18; *p* = 0.001). Taxonomic nestedness had low values in all reservoirs and ranged from 0.00 to 0.09, with no statistically significant differences (Figures 2 and 3).

Functional beta diversity was reduced in reservoirs in the middle of the cascade, with significant differences between reservoirs and between regions (beginning, middle, and end). The total component ranged from 0.00 to 0.42, and only the LA and ES reservoirs differed from each other (*F*_(2,31)_ = 3.22; *p* = 0.032), with ES presenting the highest values. However, when evaluating differences between regions, the end of the cascade presented the highest values, differing from the beginning (*F*_(2,31)_ = 3.22; *p* = 0.001). Functional turnover varied between 0.00 and 0.05, with the highest values occurring in ES and the lowest in SM, PA and LA (*F*_(6,27)_ = 3.47; *p* = 0.002; 0.039; 0.025, respectively). In terms of longitudinal regions, the lowest values, presented at the beginning and in the middle of the cascade, differentiated them from the end (*F*_(2,31)_ = 6.33; *p* = 0.012; 0.002 respectively). In turn, functional nestedness, with values between 0.00 and 0.70, showed ES with the highest values standing out from LA and SM (*F*_(6,27)_ = 2.81; *p* = 0.007; 0.004, respectively); however, the longitudinal regions did not have statistically significant differences (Figures 2 and 3).

**FIGURE 4:**
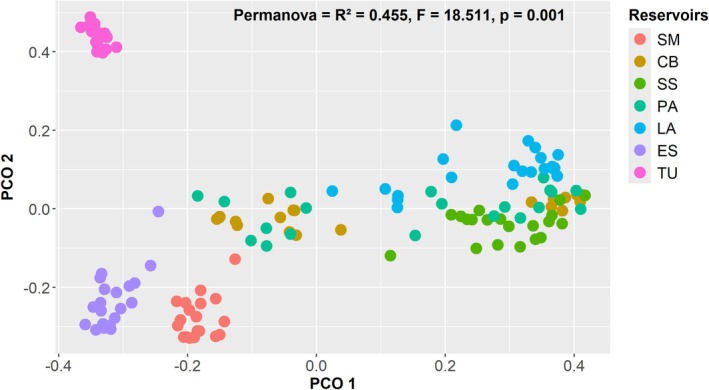
Principal coordinates analysis (PCoA) in each of the reservoirs (Serra da Mesa‐SM, Cana Brava‐CA, São Salvador‐SS, Peixe Angical‐PA, Lajeado‐LA, Estreito‐ES, and Tucuruí‐TU) in the Tocantins River.

Principal coordinate analysis (PCoA) ordination revealed a consistent pattern of differentiation in phytoplankton composition among the Tocantins River reservoirs (Figure 4). PERMANOVA confirmed significant differences between them (*R* ^2^ = 0.455, *F* = 18.511, *p* = 0.001), indicating that approximately 45% of the variation in community composition can be attributed to reservoir identity. Despite the general separation between groups, greater proximity and partial overlap were observed between the middle reservoirs (CB, SS, PA, and LA), suggesting greater similarity in the composition of their phytoplankton communities. In contrast, the reservoirs at the ends of the cascade (beginning: SM and end: ES and TU), formed well‐defined and more isolated clusters, reflecting marked differences from the others.

**FIGURE 5:**
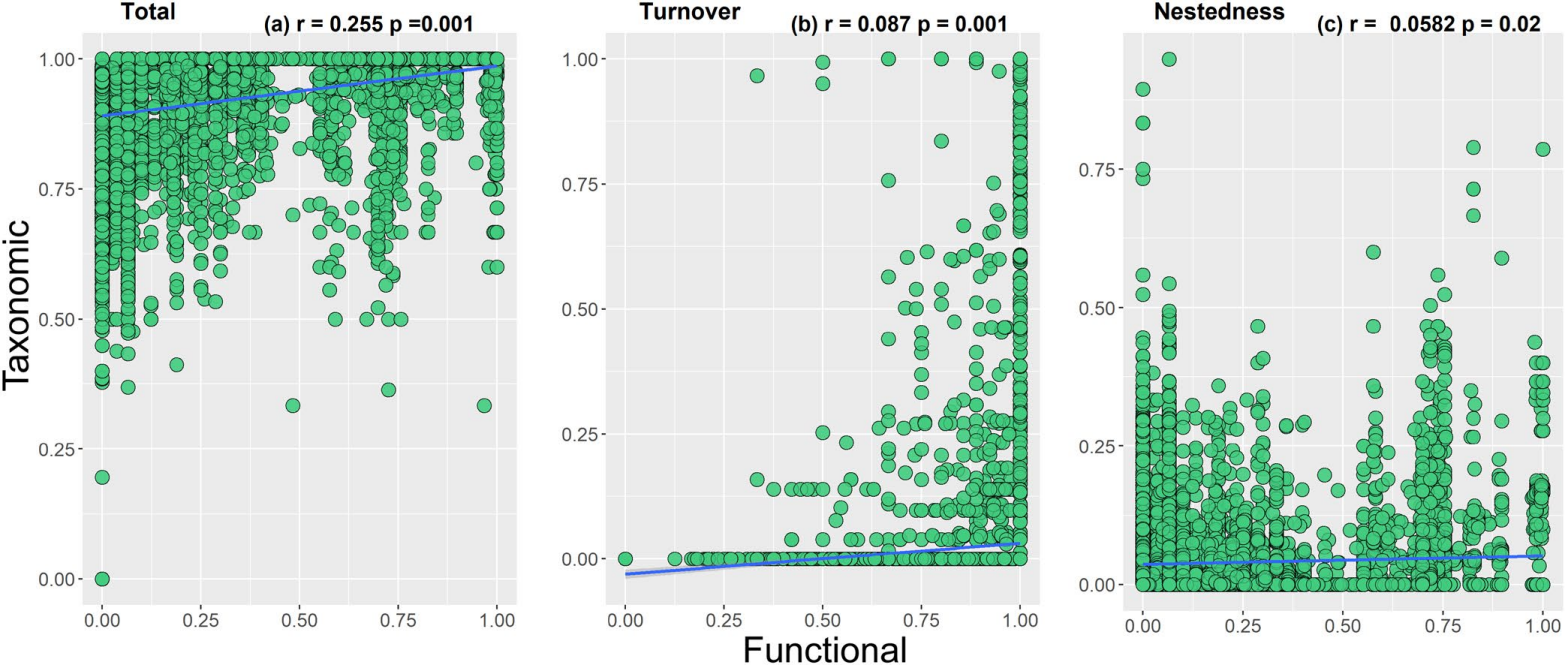
Correlation between taxonomic and functional beta diversity, and its components: (a) total, (b) turnover, and (c) nestedness. Blue line shows 95% confidence interval.

The results of the Mantel test indicated positive correlations between the components of functional and taxonomic beta diversity (Figure 5). All components showed statistically significant correlation: total beta diversity (*r* = 0.255, *p* = 0.001); turnover (*r* = 0.081, *p* = 0.001) and nestedness (*r* = 0.058, *p* = 0.001).

### 3.2 Environmental, Hydroclimatic, and Spatial Mechanisms

The results of variable selection for beta diversity components differed between the two dimensions. For taxonomic beta diversity components, the multicollinearity test and the variable selection procedure identified radiation, evaporation, flux (hydroclimatic), dissolved oxygen (DO), ammonium, total phosphorus (local environmental), in addition to the spatial components AEM 1, AEM 10, and AEM 23, AEM 26, AEM 27, AEM 4, and AEM 4 as relevant for total beta diversity. For turnover, the selected variables were radiation and flux (hydroclimatic), pH, DO, ammonium and total phosphorus (local environmental) and AEM 1, AEM 7, AEM 20, AEM 22, AEM 10, AEM 27, AEM 23, AEM 3, AEM 26, and AEM 4 (spatial). For nestedness, the hydroclimatic variables were radiation, flux, maximum temperature and evaporation; local environmental variables were temperature and conductivity, and AEM 8, AEM 10, AEM 23, AEM 11, AEM 29, AEM 27, AEM 24, AEM 13, AEM 12, AEM 5, AEM 30, AEM 4, AEM 17, AEM 19, AEM 21, AEM 9, AEM 20, AEM 22, and AEM 2 spatial variables selected. The AEM results were mostly of higher order and indicated more local effects, such as retention in a specific reservoir.

For the functional beta diversity components, flux and evaporation were selected as hydroclimatic variables, temperature, NH_3_^−^ and total phosphorus as local environmental variables, AEM 7, AEM 10, AEM 23, AEM 26, AEM 3, AEM 27, AEM 1, AEM 4 as spatial variables for total beta diversity. For turnover, the variables included evaporation and water retention time (hydroclimatic), temperature and conductivity (local environment), and AEM 24, AEM 12, AEM 8, AEM 5, AEM 21, AEM 4, AEM 29, AEM 27, AEM 7, AEM 6, AEM 9, AEM 1, and AEM 2 (spatial). For nestedness, evaporation and flux (hydroclimatic), NH_3_^−^, temperature, and total phosphorus (local environmental), and AEM 21, AEM 28, AEM 10, AEM 8, AEM 7, AEM 29, AEM 15, AEM 9, AEM 24, AEM 27, AEM 1, AEM 4, AEM 3, AEM 17, AEM 12, AEM 20, and AEM 2 (spatial) were selected. AIC, *F*, and *p* values are shown in Tables S1 and S2.

**FIGURE 6:**
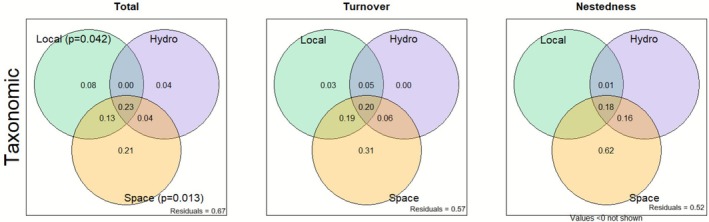
Venn diagrams for the partitioning of taxonomic beta diversity variation into three components (total, turnover, and nestedness) and the relative contributions of hydroclimatic (Hydro), local environmental (Local) and spatial (Space) variables to the explanation of variation in taxonomic beta diversity.

**FIGURE 7:**
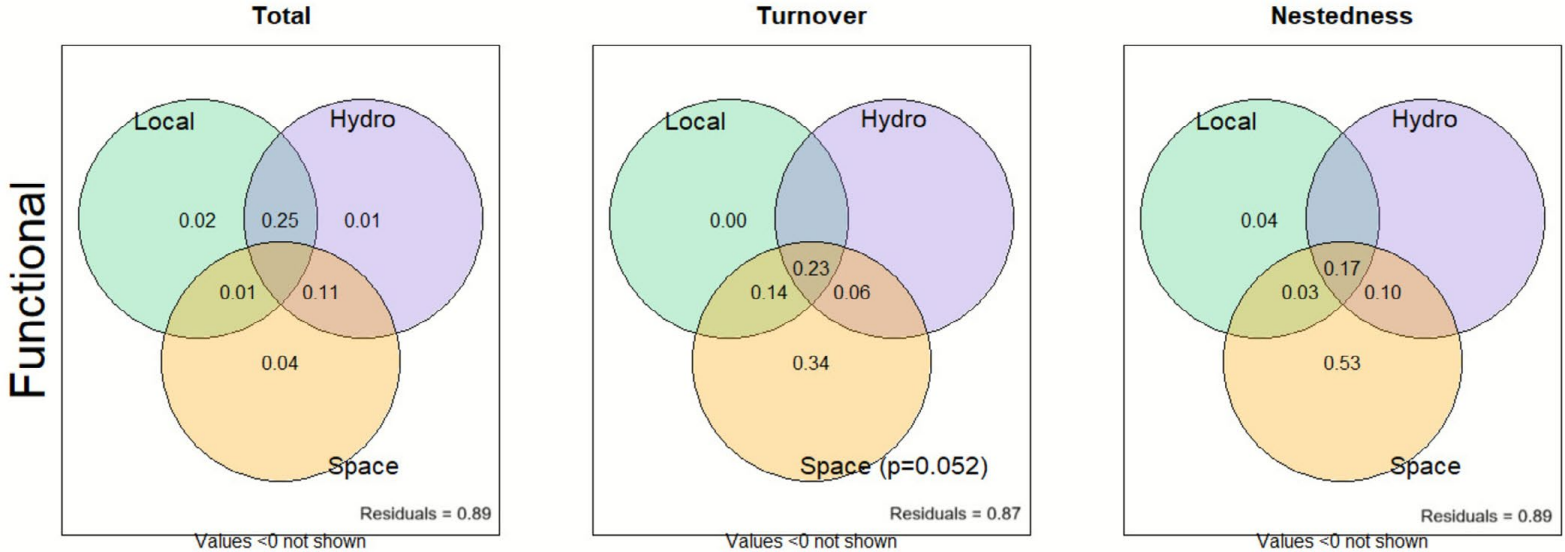
Venn diagrams for the partition of the variation in functional beta diversity into three components: total, turnover, and nestedness, and the relative contributions of hydroclimatic (Hydro), local environmental (Local) and spatial (Space) variables to explain the variation in functional beta diversity.

The results indicated that spatial processes played a predominant role in the differentiation of phytoplankton communities, while local and hydroclimatic variables had a secondary influence. Spatial variables were the main drivers of both taxonomic and functional beta diversity (Figures 6 and 7). Venn diagram analysis of taxonomic beta diversity indicated that the total component provided a significant pure explanation of 21% of the observed spatial variation. Similarly, Venn diagram analysis of functional beta diversity revealed a consistent pattern, with even more significant values for spatial variations, whose pure explanation reached 34%, particularly for the turnover component. In contrast, local and hydroclimatic variables did not provide significant pure explanations for any of the beta diversities.

## 4 Discussion

In our study, we observed that the youngest reservoir, located at the end of the cascade, presented the highest values of beta diversity, both taxonomic and functional, in the phytoplankton communities. The reservoirs located in the intermediate regions of the cascade, which are more environmentally similar and spatially closer, exhibited less taxonomic and functional variation, as well as a more similar species composition, corroborating our first hypothesis. Our results also confirmed the second hypothesis, revealing positive correlations between the components of taxonomic and functional beta diversity. Finally, we verified that variations in species composition and their functional characteristics within the phytoplankton communities were significantly influenced by spatial factors, while local hydroclimatic and environmental conditions did not show a significant influence, thus confirming our third hypothesis.

### 4.1 Age and Environmental Similarity of Cascade Reservoirs Are Responsible for Structuring Phytoplankton Beta Diversity

In our study, taxonomic and functional beta diversity were higher in the last two reservoirs (ES and TU), with the youngest reservoir in the cascade (ES) presenting greater beta diversity. This is due to recent hydrological changes resulting from the lotic–lentic transition in this environment, which changed the ecological dynamics, accelerating the colonization processes by new species, providing greater variability in species composition. New species establish themselves, while others become extinct in more distant and younger reservoirs with recently altered hydrological and environmental dynamics (da Silva et al. 2020), favoring the variability of species and functional traits. During the filling of reservoirs, the waters expand, flooding new areas and tributaries, contributing to the turnover of species and an increase in beta diversity (Poff et al. 2007; Machado et al. 2019; da Silva et al. 2020). There are not many studies on phytoplankton succession processes in young reservoirs; however, recent studies for already established reservoirs have revealed that hydrological changes, such as high water phase, mixing regime, retention time and downstream distances contribute to the succession of phytoplankton species (Xiao et al. 2011; Dunck et al. 2016; Lei et al. 2018; Lu et al. 2020).

The last reservoir (TU) was the one that presented the highest beta diversity values, after ES. This reservoir is the oldest in the cascade (formed in 1984) and the most distant, with approximately 300 km of river course free of dams. It is located in an area of high landscape biodiversity favored by transition regions between Cerrado and Amazon Rainforest. Additionally, this reservoir receives water from large tributaries, not impacted by dams, such as the Araguaia River, which has high phytoplankton beta diversity (Machado et al. 2019). Free‐flowing tributaries have been shown to be important for maintaining biodiversity for dam‐impacted rivers in Brazil (Vasconcelos et al. 2020). Another factor to be considered in this area is the high landscape diversity, which increases the availability of habitats, expanding the species niche and increasing aquatic biodiversity (Gao et al. 2004; Melo et al. 2012). The age of this reservoir also represents a factor to be addressed in the ecology of phytoplankton. Studies carried out shortly after its filling recorded high decomposition of organic matter from flooded trees and anoxia that stabilized in a short period of time (Melack 2005). More than 20 years after its formation, the Tucuruí reservoir has shown greater stability in its local environmental and ecological conditions (Straskraba et al. 1993; Silva, Rodrigues, et al. 2025). All these factors together, namely distance, landscape variability, large contributing tributaries free of dams and age, favored greater variation in species and functional traits, as found in this reservoir.

The patterns in species composition suggest that spatial proximity and greater environmental similarity between intermediate reservoirs (SS, PA, and LA), favored more similar communities. Lower values of taxonomic and functional beta diversity were found in these reservoirs. More connected communities with high dispersion are more similar in environmentally homogeneous sites (Chase 2003). In impacted aquatic ecosystems, endemic species richness may decrease, while cosmopolitan species richness may increase (Larned et al. 2010). Studies state that impacts from hydroelectric plants homogenize environmental characteristics and cause the taxonomic and functional homogenization of several communities (Algarte et al. 2016; Petsch 2016; Zhang et al. 2021; da Silva et al. 2023). On a larger scale, impacted environments accelerate species loss and reduce beta diversity (Weijters et al. 2009; Chase et al. 2019; Rolls et al. 2023), forcing functional convergence (Villéger et al. 2013). Thus, the greater environmental similarity found in the reservoirs of the middle region of the cascade led to a reduction in the taxonomic and functional dissimilarity of the phytoplankton species. In very close reservoirs, few or no tributaries enter due to the short geographic space between them, and this reduces the entry of new species (Graco‐Roza et al. 2021). Consequently environmental homogenization is greater, and leads to a scenario of biotic and functional homogenization (Olden 2006; Moyle and Mount 2007; Petsch 2016).

### 4.2 Relationship Between Taxonomic and Functional Beta Diversity

We found positive correlations between taxonomic and functional beta diversity components. We demonstrated that as species composition changed between reservoirs, functional traits of phytoplankton communities also changed, and communities with greater variation in species also showed greater variation in traits. Taxonomic groups tend to exhibit similar variation in functional trait composition across environmental gradients because they share responses to similar ecological conditions, such as changes in hydrological regimes (Heino et al. 2013). Similar results to ours were observed by other authors who attributed the positive correlation between functional beta diversity and taxonomic beta diversity to niche filtering, where only species with suitable traits remain and changes in species diversity are paired with changes in functional diversity (Perez Rocha et al. 2019; Wu et al. 2021). Furthermore, this relationship may indicate a pattern of low functional redundancy (Dunck et al. 2016). Thus, environmental filters in the cascade reservoirs of the Tocantins River led to a large variation in both species and traits.

Phytoplankton plays a key ecological role as the primary producer at the base of aquatic food webs, sustaining higher trophic levels and regulating key ecosystem processes such as nutrient cycling and energy flow (Graham et al. 2016; Chakraborty 2021; Naselli‐Flores and Padisák 2022). Therefore, changes in their taxonomic and functional beta diversity have direct implications for ecosystem functioning and resilience (Salmaso et al. 2015; Mitra et al. 2016). Communities with low redundancy may be particularly vulnerable to environmental alterations (Naeem 1998), since the loss of species can also entail the loss of unique functional traits (Cadotte et al. 2011), potentially compromising primary production and the stability of the food web. In cascading reservoirs, where hydrological regimes are strongly modified, protecting and maintaining phytoplankton diversity is essential not only for preserving biodiversity itself but also for ensuring the sustainability of ecosystem services that support fish and other aquatic organisms.

### 4.3 Relative Contributions of Environmental, Hydroclimatic and Spatial Variables to Phytoplankton Beta Diversity

Our hypothesis that spatial mechanisms would exert a greater influence than environmental and hydroclimatic factors in determining phytoplankton beta diversity, with a greater explanation for functional beta diversity, was confirmed. Spatial processes such as hydrological connectivity, position within the reservoir cascade, downstream dispersal limitation, and the spatial structure of the cascade reservoir system often structure communities more significantly than water physicochemical variables in regulated river systems (Leibold et al. 2004; Heino et al. 2015). Regulated environments lose microhabitats (Ward and Tockner 2001), and environmental variables are relatively homogeneous or exhibit little spatial variability (Rolls et al. 2023). This makes the spatial component more important (Wang et al. 2021). Furthermore, local variation, while relevant, often captures current conditions, while spatial structure reflects both dispersal processes (mass effect and dispersal limitation) and historical influences and connectivity patterns (Borcard et al. 1992). The AEM analysis revealed that most of the selected eigenvectors were of higher order, suggesting that the spatial structure of beta diversity was primarily shaped by local directional processes rather than by broad‐scale gradients along the cascade of reservoirs. This pattern indicates that fine‐scale spatial variability, such as local hydrodynamic conditions or retention effects in specific reservoirs, plays a stronger role in structuring phytoplankton beta diversity than large‐scale upstream–downstream gradients.

Although niche‐based processes contribute more to the structuring of phytoplankton communities, we demonstrated that space, represented by geographic distances, played a preponderant role in explaining the functional beta diversity of these communities. The spatial arrangement of reservoirs was a crucial factor for the functional structuring of phytoplankton communities. Studies support the idea that not only environmental mechanisms act in this structuring, but also the functional traits of the organisms and their mode of dispersal (De Bie et al. 2012; Padial et al. 2014). Thus, communities tend to organize themselves in a more similar way in nearby spaces, hydrologically connected and with similar environmental conditions (Meynard et al. 2011; Loures and Pompeu 2018; Isabwe et al. 2022). In our study, although the reservoirs are hydrologically connected, only the three reservoirs in the center of the cascade are close and similar in their environmental conditions. However, the other reservoirs are distant from each other, and the grouping of all of them in a cascade represents limits to dispersion, as the formation of environmental filters, composed of local climatic and environmental gradients along the route, selects species with functional traits adapted to each condition (Da Silva et al. 2005; Graco‐Roza et al. 2021). Functional traits of phytoplankton, such as unicellular life forms and buoyancy traits like flagella, present in some cyanobacteria and some flagellates, lead to a greater potential for dispersal, and are transported by water currents, which facilitates the colonization of new environments (Bovo‐Scomparin et al. 2013). Therefore, the distance between the cascade reservoirs is not just a geographical measurement, but a determining factor in the ecology of phytoplankton communities, directly intervening in the composition of functional traits and the functionality of the Tocantins River reservoirs.

## 5 Conclusion

Our study revealed that environmental gradients in large reservoir cascades played an important role in structuring phytoplankton beta diversity, both in taxonomic and functional terms. We demonstrated that younger and more distant reservoirs presented higher values of taxonomic and functional phytoplankton beta diversity, and that reservoirs in the intermediate regions of the cascade, close to each other and with greater environmental similarity, presented taxonomic and functional homogenization of phytoplankton. Our results indicated that spatial mechanisms, such as proximity or distances between reservoirs, have a greater influence on taxonomic and functional beta diversity. Our study highlights the importance of more efficient planning for cascade dam installations. Factors such as distances between dams, preservation of drainage microbasins, presence of dam‐free tributaries and the biomes involved can favor phytoplankton diversity in reservoirs, since they serve as sources for the entry of new species into the system. These measures contribute to increasing environmental heterogeneity and gene flow, resulting in the strengthening of local ecosystem functions. Changes in phytoplankton species composition and functional traits can compromise important ecosystem services, such as primary production, which sustains the entire trophic chain of these reservoirs, and oxygen generation, essential for both aquatic and terrestrial organisms—since some of this oxygen can be transferred to the atmosphere and used by other organisms, including humans. Another relevant ecosystem service provided by phytoplankton is pollution reduction, as these microorganisms actively participate in nutrient cycling, absorbing nitrogenous and phosphate compounds. Therefore, maintaining microalgae biodiversity in these reservoirs is fundamental to preserving the integrity and balance of the ecosystem services they offer.

## Author Contributions

**Idelina Gomes da Silva:** conceptualization (equal), data curation (equal), formal analysis (equal), methodology (equal), project administration (equal). **Bárbara Dunck:** supervision (equal), validation (equal).

## Conflicts of Interest

The authors declare no conflicts of interest.

## Supporting information


**Data S1:** ece372645‐sup‐0001‐DataS1.csv.

**Table S1:** Results of the selection of hydroclimatic, local environmental (physicochemical) and spatial (AEM) variables for components of taxonomic beta diversity. AIC, *F*, and *p* values are shown. All selected variables do not show significant multicollinearity (with variance inflation factor—vif < 5). For the meanings of the acronyms of the variables, see Table 1.
**Table S2:** Results of the selection of hydroclimatic (Hydro), local environmental (physicochemical) and spatial (AEM) variables for components of functional beta diversity. AIC, *F*, and *p* values are shown. All selected variables do not show significant multicollinearity (with variance inflation factor—vif < 5). For the meanings of the acronyms of the variables see Table 1.

## Data Availability

The data is provided as Supporting Information S1, Tables S1 and S2.
