# Supplementary material for: The Role of Habitat, Climate, and Space in the Species and Traits Variation of Phytoplankton in Large Cascade Reservoirs
Source: Ecol Evol. 2026 Jan 14;16(1):e72645. doi: 10.1002/ece3.72645 (PMC12803800; doi:10.1002/ece3.72645)
Supplement: Supplementary file 2 — Table S1: Results of the selection of hydroclimatic, local environmental (physicochemical) and spatial (AEM) variables for components of taxonomic beta diversity. AIC, F, and p values are shown. All selected variables do not show significant multicollinearity (with variance inflation factor—vif < 5). For the meanings of the acronyms of the variables, see Table 1. Table S2: Results of the selection of hydroclimatic (Hydro), local environmental (physicochemical) and spatial (AEM) variables for components of functional beta diversity. AIC, F, and p values are shown. All selected variables do not show significant multicollinearity (with variance inflation factor—vif < 5). For the meanings of the acronyms of the variables see Table 1. [file ECE3-16-e72645-s001.docx]

**Supplementary material**

**Table S1.** Results of the selection of hydroclimatic, local environmental (physicochemical) and spatial (AEM) variables for components of taxonomic beta diversity. AIC, F and p values ​​are shown. All selected variables do not show significant multicollinearity (with variance inflation factor - vif <5). For the meanings of the acronyms of the variables, see Table 1.

| Total | | | | Turnover | | | | Nestedness | | | |
| --- | --- | --- | --- | --- | --- | --- | --- | --- | --- | --- | --- |
| Variables | AIC | F | P | Variables | AIC | F | p | Variables | AIC | F | P |
| **Hydroclimatic** | | | | **Hydroclimatic** | | | | **Hydroclimatic** | | | |
| Rad | -108.28 | 5.068 | 0.050 | Rad | -89.148 | 6.601 | 0.045 | Rad | -150.1 | 2.885 | 0.052 |
| EVP | -108.68 | 4.655 | 0.030 | Flow | -83.656 | 13.19 | 0.010 | Flow | -147.1 | 5.850 | 0.040 |
| Flow | -102.60 | 11.439 | 0.005 |  |  |  |  | TempMax | -148.7 | 4.274 | 0.035 |
|  |  |  |  |  |  |  |  | EVP | -141.8 | 11.786 | 0.005 |
| **Locais** | | | | **Locais** | | | | **Locais** | | | |
| DO | -116.75 | 3.988 | 0.001 | pH | -101.22 | 3.239 | 0.050 | Temp | -140.5 | 3.151 | 0.051 |
| Ammonium | -114.48 | 6.339 | 0.005 | DO | -99.579 | 4.839 | 0.040 | Cond | -140.7 | 2.978 | 0.052 |
| TP | -107.19 | 15.029 | 0.005 | Ammonium | -100.36 | 4.066 | 0.030 |  |  |  |  |
|  |  |  |  | TP | -95.511 | 9.140 | 0.010 |  |  |  |  |
| **AEM** | | | | **AEM** | | | | **AEM** | | | |
| AEM 1 | -117.61 | 10.9026 | 0.053 | AEM 1 | -122.35 | 3.196 | 0.053 | AEM 8 | -216.2 | 3.6302 | 0.053 |
| AEM 10 | -124.61 | 4.0364 | 0.052 | AEM 7 | -121.76 | 3.6396 | 0.052 | AEM 10 | -214.1 | 4.788 | 0.050 |
| AEM 23 | -123.19 | 5.3235 | 0.040 | AEM 20 | -120.86 | 4.326 | 0.050 | AEM 23 | -213.1 | 5.323 | 0.050 |
| AEM 26 | -121.40 | 7.0102 | 0.030 | AEM 22 | -120.26 | 4.7975 | 0.035 | AEM 11 | -209.7 | 7.334 | 0.025 |
| AEM 27 | -121.22 | 7.1885 | 0.020 | AEM 10 | -118.20 | 6.4673 | 0.035 | AEM 29 | -208.1 | 8.407 | 0.020 |
| AEM 3 | -120.57 | 7.828 | 0.01 | AEM 27 | -111.75 | 12.412 | 0.015 | AEM 27 | -206.4 | 9.515 | 0.020 |
| AEM 4 | 1 -114.16 | 4.8495 | 0.005 | AEM 3 | -114.19 | 10.033 | 0.010 | AEM 24 | -196.8 | 17.239 | 0.015 |
|  |  |  |  | AEM 23 | -111.94 | 12.218 | 0.010 | AEM 13 | -190.7 | 23.343 | 0.010 |
|  |  |  |  | AEM 26 | -116.03 | 8.3455 | 0.005 | AEM 12 | -203.3 | 11.743 | 0.005 |
|  |  |  |  | AEM 1 | -107.63 | 16.846 | 0.005 | AEM 5 | -200.8 | 13.783 | 0,005 |
|  |  |  |  | AEM 4 | -94.65 | 34.908 | 0.005 | AEM 30 | -198.3 | 15.891 | 0.005 |
|  |  |  |  |  |  |  |  | AEM 4 | -197.8 | 16.267 | 0.005 |
|  |  |  |  |  |  |  |  | AEM 17 | -191.9 | 22.0126 | 0.005 |
|  |  |  |  |  |  |  |  | AEM 19 | -190.1 | 24.041 | 0.005 |
|  |  |  |  |  |  |  |  | AEM 21 | -185.4 | 29.587 | 0.005 |
|  |  |  |  |  |  |  |  | AEM 9 | -181.8 | 34.467 | 0.005 |
|  |  |  |  |  |  |  |  | AEM 20 | -176.3 | 43.111 | 0.005 |
|  |  |  |  |  |  |  |  | AEM 22 | -169.0 | 56.677 | 0.005 |
|  |  |  |  |  |  |  |  | AEM 2 | -155.0 | 92.612 | 0.005 |

**Table S2.** Results of the selection of hydroclimatic (Hydro), local environmental (physicochemical) and spatial (AEM) variables for components of functional beta diversity. AIC, F and p values ​​are shown. All selected variables do not show significant multicollinearity (with variance inflation factor- vif <5). For the meanings of the acronyms of the variables see Table 1.

| Total | | | | Turnover | | | | Nestedness | | | |
| --- | --- | --- | --- | --- | --- | --- | --- | --- | --- | --- | --- |
| Variables | AIC | F | P | Variables | AIC | F | p | Variables | AIC | F | P |
| **Hydroclimatic** | | | | **Hydroclimatic** | | | | **Hydroclimatic** | | | |
| Flow | -8.570 | 4.590 | 0.040 | EVP | -187.85 | 5.517 | 0.035 | EVP | -10.88 | 4.362 | 0.052 |
| EVP | -8.547 | 4.614 | 0.040 | TRH | -180.17 | 14.77 | 0.005 | Flow | -10.43 | 4.832 | 0.040 |
| **Locais** | | | | **Locais** | | | | **Locais** | | | |
| Temp | -7.599 | 3.090 | 0.052 | Temp | -186.78 | 5.920 | 0.035 | NH_3_^-^ | -9.470 | 4.234 | 0.050 |
| NH_3_^-^ | -6.905 | 3.771 | 0.053 | Cond | -176.21 | 19.37 | 0.005 | Temp | -10.29 | 3.415 | 0.040 |
| TP | -0.283 | 11.03 | 0.005 |  |  |  |  | TP | -2.46 | 12.070 | 0.005 |
| **AEM** | | | | **AEM** | | | | **AEM** | | | |
| AEM 7 | -127.52 | 2.794 | 0.054 | AEM 24 | -213.2 | 3.88 | 0.053 | AEM 21 | -57.841 | 4.943 | 0.055 |
| AEM 10 | -125.71 | 4.315 | 0.050 | AEM 12 | -212.68 | 4.30 | 0.052 | AEM 28 | -59.555 | 3.914 | 0.054 |
| AEM 23 | -124.15 | 5.691 | 0.050 | AEM 8 | -212.7 | 4.24 | 0.050 | AEM 10 | -59.636 | 3.867 | 0.054 |
| AEM 26 | -122.21 | 7.494 | 0.015 | AEM 5 | -211.9 | 4.86 | 0.050 | AEM 8 | -58.266 | 4.683 | 0.045 |
| AEM 3 | -121.30 | 8.369 | 0.015 | AEM 21 | -211.7 | 4.95 | 0.045 | AEM 7 | -56.768 | 5.615 | 0.025 |
| AEM 27 | -122.01 | 7.684 | 0.010 | AEM 4 | -210.2 | 6.08 | 0.025 | AEM 29 | -52.826 | 8.272 | 0.020 |
| AEM 1 | -118.11 | 11.65 | 0.010 | AEM 29 | -209.9 | 6.37 | 0.025 | AEM 15 | -54.934 | 6.813 | 0.015 |
| AEM 4 | -114.41 | 15.87 | 0.005 | AEM 27 | -208.2 | 7.72 | 0.025 | AEM 9 | -51.132 | 9.513 | 0.015 |
|  |  |  |  | AEM 7 | -211.1 | 5.45 | 0.020 | AEM 24 | -50.750 | 9.80 | 0.015 |
|  |  |  |  | AEM 6 | -208.1 | 7.73 | 0.010 | AEM 27 | -49.720 | 10.59 | 0.005 |
|  |  |  |  | AEM 9 | -202.1 | 13.1 | 0.005 | AEM 1 | -47.150 | 12.68 | 0.005 |
|  |  |  |  | AEM 1 | -200.2 | 15.0 | 0.005 | AEM 4 | -45.690 | 13.94 | 0.005 |
|  |  |  |  | AEM 2 | -186.9 | 31.8 | 0.005 | AEM 3 | -43.058 | 16.35 | 0.005 |
|  |  |  |  |  |  |  |  | AEM 17 | -42.645 | 16.74 | 0.005 |
|  |  |  |  |  |  |  |  | AEM 12 | -41.018 | 18.35 | 0.005 |
|  |  |  |  |  |  |  |  | AEM 20 | -32.096 | 28.65 | 0.005 |
|  |  |  |  |  |  |  |  | AEM 2 | -15.236 | 57.32 | 0.005 |
